# Tunable Dynamic Black Phosphorus/Insulator/Si Heterojunction Direct-Current Generator Based on the Hot Electron Transport

**DOI:** 10.34133/2019/5832382

**Published:** 2019-11-15

**Authors:** Yanghua Lu, Sirui Feng, Runjiang Shen, Yujun Xu, Zhenzhen Hao, Yanfei Yan, Haonan Zheng, Xutao Yu, Qiuyue Gao, Panpan Zhang, Shisheng Lin

**Affiliations:** ^1^College of Microelectronics, College of Information Science and Electronic Engineering, Zhejiang University, Hangzhou 310027, China; ^2^State Key Laboratory of Modern Optical Instrumentation, Zhejiang University, Hangzhou 310027, China

## Abstract

Static heterojunction-based electronic devices have been widely applied because carrier dynamic processes between semiconductors can be designed through band gap engineering. Herein, we demonstrate a tunable direct-current generator based on the dynamic heterojunction, whose mechanism is based on breaking the symmetry of drift and diffusion currents and rebounding hot carrier transport in dynamic heterojunctions. Furthermore, the output voltage can be delicately adjusted and enhanced with the interface energy level engineering of inserting dielectric layers. Under the ultrahigh interface electric field, hot electrons will still transfer across the interface through the tunneling and hopping effect. In particular, the intrinsic anisotropy of black phosphorus arising from the lattice structure produces extraordinary electronic, transport, and mechanical properties exploited in our dynamic heterojunction generator. Herein, the voltage of 6.1 V, current density of 124.0 A/m^2^, power density of 201.0 W/m^2^, and energy-conversion efficiency of 31.4% have been achieved based on the dynamic black phosphorus/AlN/Si heterojunction, which can be used to directly and synchronously light up light-emitting diodes. This direct-current generator has the potential to convert ubiquitous mechanical energy into electric energy and is a promising candidate for novel portable and miniaturized power sources in the in situ energy acquisition field.

## 1. Introduction

Since the formal discovery of heterojunctions in the mid-20th century [1, 2], solid-state electronics have been considerably advanced, which has laid the foundation for the modern information society [3]. As the carrier dynamic transport between heterojunctions can be designed through band gap engineering of semiconductors [4], the van der Waals heterojunction is applicable to many advanced electronic and optoelectronic fields [5], such as blue light-emitting diodes [6, 7], photodetectors [8, 9], solar cells [10, 11], transistors [12], and other emerging applications. However, the new development of intelligent and wearable electronic heterojunction devices puts forward new requirements for in situ electric energy acquisition technology [13, 14]. Since the electromagnetic generator propelled mankind into the age of electricity, electric energy has been driving the development of human society for centuries [15]. As age advances, it is Increasingly important to create a lightweight and miniaturized electric generator with high power density output, which is still the technical difficulty of conventional generators [16–18]. As all the heterojunction devices are based on static heterojunctions, why the heterojunction should be static rather than dynamic is quite an intuitive question. This question possibly points to the development of the abovementioned in situ energy acquisition technology, as there has been little exploration of dynamic heterojunctions in both the information and energy fields.

Recently, we have designed the creative systematic design of dynamic Schottky diodes, in which the sliding of metals over semiconductors can produce a direct-current electricity output [19–21]. Some similar phenomena based on semiconductors have also been dedicated to converting mechanical energy into electricity, which they attribute to the triboelectricity effect [22–26]. In particular, we have proposed the physical picture of the dynamic Schottky diode based on the rebounding effect under the ultrahigh built-in electric field at the interface, which can break the balance between the drift current and diffusion current and generate an electrical output [19, 20]. The mechanism of the direct-current generator based on dynamic junctions should be thoroughly explored in the improvement of its performance. Thus, a dynamic semiconductor/semiconductor structure [27], especially a “dynamic heterojunction” defined by moving one semiconductor over another semiconductor with a different Fermi level, should be thoroughly investigated to prove our distinctive current generation mechanism. As the Fermi level difference between semiconductors, which is much larger than that between a metal and a semiconductor, can be designed through heterointegration, the output voltage is expected to be largely improved. In this work, we indicate that the hot carrier kinetic process in a dynamic heterojunction is of critical importance for increasing the output voltage [28, 29], which also confirms the proposed physical picture of the rebounding effect. In particular, black phosphorus, a representative and emerging layer semiconductor, has recently received great attention from researchers for its ultrahigh hole mobility, unique intrinsic anisotropy, tunable band gap, and theoretical capacity [30–32], which can be used to design this hot carrier kinetic process and increase the interface rebounding effect. Herein, a dynamic generator based on a black phosphorus/Si heterojunction has been achieved, where both black phosphorus and silicon are semiconductors. By moving black phosphorus over silicon, the carriers are reflected under the ultrahigh interface barrier in the dynamic heterojunction, which breaks the balance of the drift current and diffusion current. The reflected electrons and holes can be accelerated by the huge built-in electric field formed by black phosphorous and silicon and become high-energy hot carriers. These hot carriers can be located at the quasi Fermi level higher than the static equilibrium Fermi level of the semiconductor; thus, the output voltage can be higher than the static Fermi level difference of the two semiconductors, leading to a high voltage output. Meanwhile, as a result of the ultrahigh hole mobility and unique intrinsic anisotropy arising from the lattice structure of black phosphorus [33, 34], the reflected hot carriers can be effectively collected by the electrodes with limited energy degradation, leading to a much higher current output as well as a higher power conversion efficiency.

Semiconductor physics is developed from deep mechanics at the atomic level. To quantitatively demonstrate the physical mechanism of the hot electron transport due to the interface barrier, an ultrathin dielectric layer is inserted into the dynamic heterojunction interface. This hot carrier dynamic transport process between semiconductors can be delicately designed through the interface band gap engineering. The voltage output can be numerically adjusted according to the valence band difference between the semiconductors and dielectrics. By inserting a graphene layer with a zero band gap, the effect of triboelectricity is eliminated as the unshielded built-in electric field and unchanged voltage output, indicating the validity of our unique physical mechanism. By inserting an insulating layer functioning as an interface high barrier between two semiconductors, the barrier height can be enhanced and the rebounding effect can be stimulated [35]. In this work, dynamic semiconductor-insulator-semiconductor heterojunction generators with different types of insulating layers are explored. Under this severely bounding-back and acceleration effect of the large interface electric field, hot electrons will still transfer across the interface through the tunneling or hopping effect [36, 37], leading to an electrical output. In particular, a dynamic heterojunction generator with a high open-circuit voltage (*V*_oc_) of 6.1 V, a high short-circuit density (*J*_sc_) of 124.0 A/m^2^ (12.4 mA/cm^2^), a high power density of 201.0 W/m^2^, and a high energy-conversion efficiency of 31.4% has been realized based on the dynamic black phosphorus/AlN/Si heterojunction. The voltage output of the black phosphorus/AlN/Si heterojunction is one order of magnitude larger than that of the dynamic metal/semiconductor junction and can be used to directly and synchronously light up commercial light-emitting diodes (LEDs) without any external rectifying circuits or energy storage units. Such advantages favor the great potential of this generator for practical application in the near future. This dynamic heterojunction generator also exhibits an ultrahigh current density, which is ~10^3^ times higher than triboelectric nanogenerators and ~10^4^ times higher than piezoelectric nanogenerators [17, 18], indicating its unique integration and application potential in the in situ energy acquisition field. We believe that this dynamic heterojunction generator will certainly open a new avenue for the physical research of dynamic heterojunctions, as well as be a promising substitute for portable and integrated energy chips, in parallel with the development of integrated information chips.

## 2. Results and Discussion

**Figure 1:**
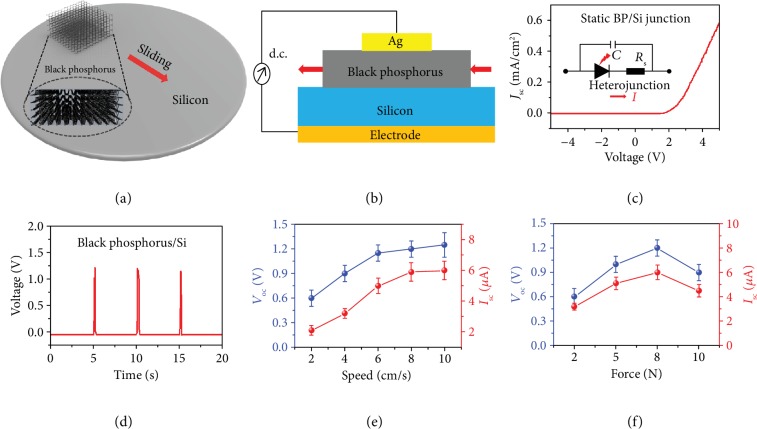
Experimental design and results of the dynamic black phosphorus/Si generator. (a) Schematic illustration of the dynamic black phosphorus/Si generator. Inset: the lattice of black phosphorus. (b) The structure of the black phosphorus/Si generator. (c) *J*-*V* curve of the static black phosphorus/Si junction with an 8.0 N force. The contact area is 0.05 mm^2^. Inset: the circuit diagram of the static black phosphorus/Si junction. (d) *V*_oc_ of the dynamic black phosphorus/Si generator under the linearly reciprocating mode with an 8.0 N force and a speed of 8.0 cm/s. (e) *V*_oc_ and *I*_sc_ of the dynamic black phosphorus/Si generator with different moving speeds and an 8.0 N force exerted on the junction. (f) *V*_oc_ and *I*_sc_ of the dynamic black phosphorus/Si generator with different forces exerted and a speed of 8.0 cm/s.

The lattice of black phosphorus is composed of six-membered rings of interconnected atoms, each of which is connected to three others, as shown in the insert of Figure 1(a) as well as in [Supplementary-material supplementary-material-1] [33, 34]. The unique intrinsic anisotropy arising from the lattice structure produces extraordinary electronic, transport, and mechanical properties that can be exploited in the design of new devices [38], especially for our dynamic heterojunction generator. A three-dimensional diagram and a schematic structure of the dynamic black phosphorus/Si generator are illustrated in Figure 1(a) and (b), respectively, where the black phosphorus simply moves over the silicon substrate. The static black phosphorus/Si heterojunction formed by the external mechanical press shows excellent rectification behavior with a low leakage current and a high threshold voltage greater than 1.0 V (Figure 1(c)). The equivalent circuit diagram is shown in the insert of Figure 1(c), which consists of an internal resistance, an equivalent capacitance, and an ideal diode. This heterojunction consists of silicon and black phosphorus, whose work functions are 5.12 eV and 4.30 eV, respectively (the calculation is given in the supporting information). As the work function of black phosphorus is less than that of the Si substrate [39], electrons will diffuse into the silicon, and holes will diffuse into the black phosphorus; thus, a built-in electric field will be formed between the black phosphorus and Si substrate in the static state, leading to a rectification characteristic.

When the black phosphorus is dragged along the surface of the Si wafer with a contact area of 0.05 mm^2^ (0.1 mm × 0.5 mm), a maximal *V*_oc_ up to 1.2 V is observed, as shown in Figure 1(d). As the Fermi level difference between semiconductors is much larger, the threshold voltage of the heterojunction is higher than that of a Schottky diode, leading to a much larger voltage output of the dynamic heterojunction generator [40]. A peak *I*_sc_ of up to 6.2 *μ*A is measured, and *J*_sc_ up to 124.0 A/m^2^ can be calculated accordingly, which is three or four orders of magnitude higher than those reported for polymer-based nanogenerators [17, 18]. The relationship between *V*_oc_/*I*_sc_ and the lateral movement speed is explored, by which we find that *V*_oc_ and *I*_sc_ increase as the moving speed increases until the moving speed reaches 8.0 cm/s (Figure 1(e)). Furthermore, the dependence of *V*_oc_/*I*_sc_ on the force applied on the black phosphorus is also explored, and a force of 8.0 N is found to be the optimal choice (Figure 1(f)). Thus, the speed of 8.0 cm/s and the force of 8.0 N are adopted in our experiments, where the dynamic heterojunction generator has the optimal junction characteristics. The force and speed are controlled with a customized system as shown in [Supplementary-material supplementary-material-1]. Notably, this direct current synchronously generates a voltage, which is completely different from triboelectric nanogenerators that only output an alternating current [19]. The triboelectricity utilizes the displacement current in the Maxwell equation, which is generated by the periodic charge-discharge process of carriers and cannot freely flow through the insulating dielectric materials; thus, a current cannot be synchronously generated with the voltage, and no continuous direct current is generated [17].

**Figure 2:**
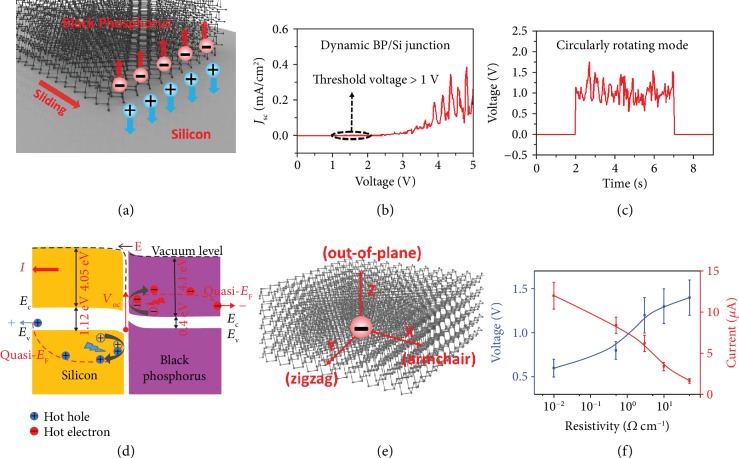
The physical mechanism of the dynamic heterojunction generator. (a) Schematic diagram of the dynamic black phosphorus/Si generator. (b) *J*-*V* curve of the dynamic black phosphorus/Si junction with an 8.0 N force. The threshold voltage is larger than 1.0 V. (c) *V*_oc_ of the dynamic black phosphorus/Si generator under the circularly rotating mode with an 8 N force and a speed of 8.0 cm/s. (d) Band diagram of the dynamic black phosphorus/Si generator. (e) The unique intrinsic anisotropy of black phosphorus arising from the lattice structure. (f) Voltage and current output of the dynamic black phosphorus/Si generator with a different Si substrate resistivity.

For this black phosphorus/Si junction, the drift and diffusion electron currents can be described as follows [41]:

(1) $$\begin{array}{c} J_{n}^{\text{drift}}=qnu_{n}E,\\ J_{n}^{\text{diff}}=qD_{n}\nabla n,\\ J_{n}=J_{n}^{\text{drift}}+J_{n}^{\text{diff}}=qnu_{n}E+qD_{n}\nabla n, \end{array}$$

where *J*_*n*_^drift^, *J*_*n*_^diff^, *J*_*n*_, *μ*_*n*_, and *D*_*n*_ are the drift electron current, diffusion electron current, electron current density, electron mobility, and electron diffusion coefficient of black phosphorus and Si, respectively. *q* is the elementary electric charge, *n* is the position-dependent electron density in black phosphorus and Si, and *E* is the built-in electric field. According to equation (1), the electron current density of the black phosphorus/Si junction consists of *J*_*n*_^drift^ and *J*_*n*_^diff^. At the static black phosphorus/Si heterojunction, *J*_*n*_^diff^ will quickly balance with *J*_*n*_^drift^, which are in the opposite directions and offset each other. When the black phosphorus moves along the silicon substrate, destruction of the heterojunction at the rear part and establishment at the front part will subsequently occur, breaking the symmetry of the drift-diffusion currents. Thus, electrons and holes are reflected to the black phosphorus and silicon, respectively, under the ultrahigh localized built-in electric field in the dynamic heterojunction, generating hot electrons and holes with high energy, which is the underlying physical picture of this dynamic heterojunction generator. These rebounding hot electrons and holes will increase the *J*_*n*_^drift^ and decrease the *J*_*n*_^diff^, generating the current output in the dynamic heterojunction.  Figure 2(a) shows a schematic illustration of the movement process of carriers at the black phosphorus/Si interface. Furthermore, as shown in the rectification characteristic of the dynamic black phosphorus/Si junction from -5 V to 5 V (Figure 2(b)), there are some oscillations in the *J*-*V* curve as the voltage increases, indicating variation of the interfacial built-in electric field of the heterojunction. Compared with the *J*-*V* curve of the static black phosphorus/Si junction, the current response of the dynamic heterojunction in the bias voltage is decreased with the generation of a current output, indicating the destruction of the heterojunction during the movement. The sustained direct current under the circularly rotating mode is shown in Figure 2(c), indicating our unique mechanism based on semiconductor physics of continuous bounding-back hot carriers, which is totally different from that of triboelectric generators. The periodical current oscillation that superposes on this sustained direct-current output is attributed to the variation of the force exerted on the dynamic heterojunction under the circularly rotating mode.

The detailed transport of the rebounding carriers and the band gap alignment between black phosphorus and silicon are shown in Figure 2(d). A built-in electric field is generated as a result of electron diffusion from black phosphorus to Si due to the Fermi level difference. Under the effect of the built-in electric field from black phosphorus to the Si substrate, the rebounding effect-generated hot electrons and holes will be continuously collected by the ohmic contacts of black phosphorus and Si, respectively ([Supplementary-material supplementary-material-1]). The dynamic equilibrium is achieved, and hot carriers can be located at the quasi-Fermi level higher than the static equilibrium Fermi level of the semiconductor, leading to a high voltage output. On the other hand, the intrinsic anisotropy of black phosphorus arising from the lattice structure produces extraordinary electronic, transport, and mechanical properties that can be exploited in our dynamic heterojunction generator. As shown in Figure 2(e), this unique intrinsic anisotropy produces more hot electrons in black phosphorus, leading to a higher performance. To further demonstrate the tunable direct-current generator based on dynamic heterojunction, silicon with a different resistivity is also explored. As shown in Figure 2(f), the average voltage and current output of the dynamic black phosphorus/Si generator with a different Si substrate resistivity are 0.6/0.8/1.2/1.3/1.4 V and 12.1/8.4/6.2/3.4/1.7 *μ*A, respectively. Attributed to the important role of the interface built-in electric field, an increase in the carrier concentration of the silicon wafer leads to a higher current output but a lower voltage output. With increasing carrier concentration, the built-in electric field of the black phosphorus/Si heterojunction is enhanced, leading to more electrons and holes rebounding to the semiconductors and increasing the current output. However, the increased carrier transfer between black phosphorus and Si will decrease the barrier height of the black phosphorus/Si heterojunction, leading to a decrease in the voltage output.

Recently, light and temperature have been shown to play important roles in the electron transfer of nanoscale contact electrification between a metal and an insulator [42, 43]. To eliminate the effect of illumination and temperature changes in our dynamic heterojunction, several experiments on the relationship between *V*_oc_ and the illumination and temperature of the environment are explored. *V*_oc_ exhibits limited changes in different illumination and temperature environments ([Supplementary-material supplementary-material-1] and [Supplementary-material supplementary-material-1]), indicating that the light and heat effects are not main factors here. We attribute this phenomenon to the nonoverlapping space of the light-induced hot electrons and mechanical energy-induced hot electrons. As a semiconductor with high light absorption, the black phosphorus block will absorb the incident light in the upper surface, generating hot electrons outside the depletion layer, which cannot be effectively collected by the interface built-in electric field.

**Figure 3:**
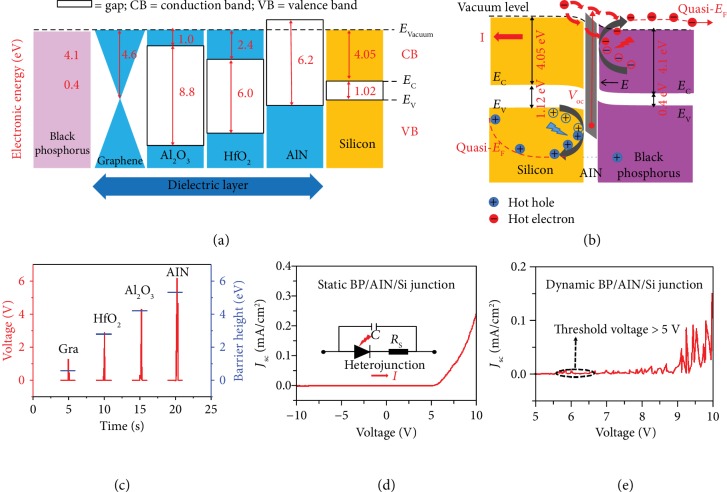
Tunable dynamic black phosphorus/dielectric layer/Si junctions based on hot electron transport. (a) One-dimensional band alignment of the conduction and valence band edges for various dielectric layers (graphene, HfO_2_, Al_2_O_3_, and AlN) and semiconductors (Si and black phosphorus). (b) Band diagram of the dynamic black phosphorus/AlN/Si generator. (c) *V*_oc_ of the dynamic heterojunction generator with different interface barrier heights. The graphene layer is a monolayer, and its insulating layer is as thin as 10 nm. The generator works under the linearly reciprocating mode with an 8.0 N force and a speed of 8.0 cm/s. (d and e) *J*-*V* curve of the static and dynamic black phosphorus/AlN/Si junction with an 8.0 N force. Inset: the circuit diagram of the static black phosphorus/AlN/Si junction. The threshold voltage is greater than 5.0 V.

Furthermore, the output voltage of dynamic heterojunction can be delicately adjusted and enhanced with the interface energy level engineering of inserting dielectric layers. Various interface dielectric layers are added to the interface of the dynamic black phosphorus/Si heterojunction, which can further testify for the proposed mechanism based on semiconductor physics and reveal the hot carrier kinetic process in the dynamic heterojunction. For the proposed mechanism, the voltage output should be delicately adjusted and enhanced with an increasing interface barrier height of the dynamic heterojunction. Thus, a dynamic semiconductor-dielectric-semiconductor sandwich heterojunction structure generator is fabricated by inserting graphene/HfO_2_/Al_2_O_3_/AlN into the interface of black phosphorus and the Si substrate. The detail one-dimensional band alignments of the energy band structure for various dielectric layers (graphene, HfO_2_, Al_2_O_3_, and AlN) and semiconductors (Si and black phosphorus) are shown in Figure 3(a) [44, 45]. The conduction/valence bands of the insulators are much higher/lower than those of black phosphorus and Si, and the band gaps are much larger than those of both black phosphorus and Si. The barrier height of the black phosphorus/insulator/Si junctions has been largely enhanced compared with the black phosphorus/Si junction, among which the AlN layer has the highest barrier height. As shown in the band diagram of the dynamic black phosphorus/AlN/Si heterojunction (Figure 3(b)), the electron and hole transfer is largely suppressed by the insertion of the 10 nm AlN layer, which acts as a barrier layer in the interlayer of the black phosphorus/Si junction. With an increase of the barrier height, the diffusing electrons and holes can be more effectively rebounded to black phosphorus and silicon, generating more hot carriers that move toward the ohmic contact of the heterojunction. The one-dimensional relative band structure and rectification characteristic of the black phosphorus/AlN/Si junction are shown in [Supplementary-material supplementary-material-1].

The detailed relationship between the voltage output for various dielectric layers (graphene, HfO_2_, Al_2_O_3_, and AlN) and the interface barrier height is shown in Figure 3(c). The average voltage output of the various dynamic black phosphorus/insulator/Si heterojunctions and the interface barrier height between Si and the graphene/HfO_2_/Al_2_O_3_/AlN layer are 1.2/2.9/4.3/6.1 V and 0.6/2.8/4.2/5.3 eV, respectively, indicating that the voltage output is tunable by inserting different dielectric layers into the interface of dynamic heterojunctions. With the insertion of the monolayer graphene with a zero band gap, the interface built-in electric field is almost unchanged, leading to the voltage output of the dynamic black phosphorus/Si junction with and without the graphene layer being the same. However, the voltage output after inserting one graphene layer into the contact interface must be changed if triboelectricity has been produced in the dynamic heterojunction, as the interface carrier capacity of heterojunction is completely changed. Similar voltages with and without the graphene layer indicate the validity of our unique physical mechanism based on semiconductor physics, verifying that triboelectricity is not the main mechanism of our dynamic heterojunction. It is noteworthy that the voltage is largely enhanced and even larger than the interface barrier height between the semiconductor and dielectric layer, indicating that the hot electrons play a key role in the voltage output of the dynamic heterojunction. This hot electron mechanism is the unique result of the rebounding effect in the dynamic heterojunction, which is self-consistent and can explain the experimental phenomenon. And under the severely bounding-back and acceleration effect of the large interface electric field (*E* ≈ 6 × 10^6^ V/cm), hot electrons will still transfer across the interface through the tunneling or hopping effect [36, 37]. Furthermore, the AlN layer with negative electron affinity exhibits the highest barrier height, which will achieve the highest voltage output according to the abovementioned unique physical mechanism. The voltage output of this black phosphorus/AlN/Si junction is as large as 6.1 V, which is much larger than the interface barrier height between the Si and AlN layer, indicating the hot electron transport through the AlN layer. The rectification characteristics of the static and dynamic black phosphorus/AlN/Si junction from -10 V to 10 V are shown in Figure 3(d) and (e), respectively. It is obvious that the threshold voltage of the black phosphorus/AlN/Si junction is much higher than 5.0 V, proving the increase of the barrier height and built-in electric field of the heterojunction, whose voltage output is high enough to light up commercial LEDs without any external rectifying circuits or energy storage units directly and synchronously.

**Figure 4:**
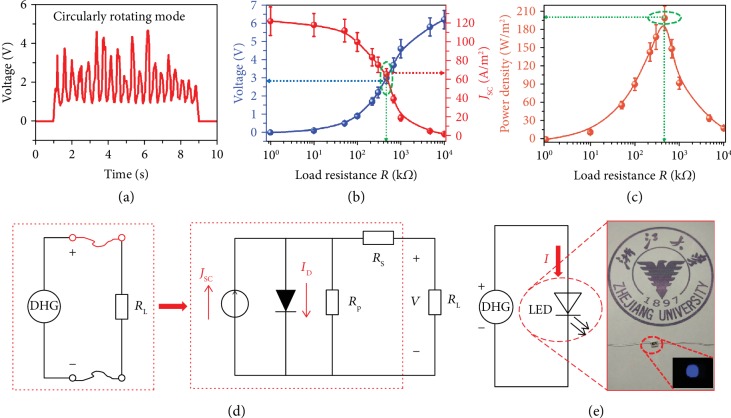
The power output measurement and potential practical application of the dynamic black phosphorus/AlN/Si generator. (a) *V*_oc_ of the dynamic black phosphorus/AlN/Si generator under the circularly rotating mode with an 8.0 N force and a speed of 8.0 cm/s. (b) Peak *V*_oc_ and *J*_sc_ output. (c) Power density output of the dynamic black phosphorus/AlN/Si generator as a function of electrical load *R*. (d) Circuit diagram and equivalent circuit of the dynamic heterojunction under load working condition. (e) Circuit diagram of the LED lighting experiment and pictures taken from a video to show the luminance of an LED powered by our dynamic heterojunction generator.

Finally, the voltage output of this black phosphorus/AlN/Si junction under the circularly rotating mode is explored, which is sustainable and continuously generated, as shown in Figure 4(a). The fluctuation of the continuous voltage output is caused by the dynamic process of the establishment and destruction of the depletion layer in this dynamic black phosphorus/AlN/Si junction and the force change of our rotating equipment. To demonstrate its potential in practical applications, we measure the power output of the dynamic black phosphorus/AlN/Si heterojunction generator as a function of electrical load *R*. As shown in Figure 4(b), the voltage and current output as a function of electrical load *R* are measured. With increasing load resistance, an increasing voltage but decreasing current can be achieved. The power density output as a function of electrical load *R* is accordingly calculated, among which a peak power density of 201.0 W/m^2^ can be achieved when the R is as high as 450 kΩ (Figure 4(c)). This resistance is equal to the internal resistance of this black phosphorus/AlN/Si heterojunction. The equivalent circuit diagram of the dynamic heterojunction is analyzed accordingly, which consists of the internal resistance (*R*_s_ + *R*_p_), a leakage current (*I*_D_), and a load resistance (*R*_L_), as shown in Figure 4(d).

The power conversion efficiency of the dynamic heterojunction generator can be expressed as follows:

(2) $$\begin{array}{c} \text{PCE}=\frac{P_{\max}}{P_{\text{in}}}=\frac{V_{\text{oc}}\times J_{\text{sc}}\times \text{FF}}{P_{\text{in}}}=\frac{V_{\max}\times J_{\max}}{P_{\text{in}}}=\frac{V_{\max}\times J_{\max}}{F\times v}, \end{array}$$

where *V*_oc_ and *J*_sc_ are the open-circuit voltage and short-circuit current density of the generator. And *V*_max_ and *J*_max_ are the working voltage and current density of the generator. FF is the fill factor, *F* is the friction in the heterojunction interface, and *v* is the relative moving speed. *V*_max_ and *J*_max_ are calculated with the average working voltage and current density of the direct-current generator in 5.0 s. According to the power output and mechanical energy input, the energy-conversion efficiency of the dynamic black phosphorus/AlN/Si junction generator can be calculated to be as high as 31.4%, which is much higher than that of the dynamic Schottky diode generator (the details are shown in the supporting information). The power density and energy-conversion efficiency are much higher than that of the dynamic Schottky diode generator, which is attributed to the unique intrinsic anisotropy of black phosphorus and higher Fermi level difference leading to more reflected hot carriers being effectively collected by the electrodes with limited energy degradation. As our dynamic black phosphorus/AlN/Si heterojunction generator can provide a nearly 6.1 V open-circuit voltage and 124.0 A/m^2^ short-circuit current density, it can be used to provide sufficiently high work voltage for directly lighting a blue LED without the limitation of external rectifying circuits or energy storage units. The detailed experimental model and captured optical pictures are shown in [Supplementary-material supplementary-material-1]. The circuit diagram of the LED lighting experiment and a picture of the LED powered by our dynamic black phosphorus/AlN/Si heterojunction generator are shown in Figure 4(e). In particular, the blue-light LED is synchronously and continuously lighted up with our mechanical movement, indicating the generation of a continuous direct current, which is achieved by the abovementioned unique physical mechanism. This direct-current generator has the potential to convert ubiquitous mechanical energy into a constant current output and provides a feasible way to satisfy the increasing demand of in situ energy acquisition technology. We believe that this dynamic heterojunction generator will be a promising substitute for portable and integrated energy chips in parallel with the development of integrated information chips.

## 3. Conclusion

This work has demonstrated a tunable high-performance direct-current generator based on a dynamic heterojunction, which can output a voltage as large as 1.2 V using a black phosphorus/silicon heterojunction as the representative device. The intrinsic anisotropy of black phosphorus arising from the lattice structure produces extraordinary electronic, transport, and mechanical properties exploited in our dynamic heterojunction generator. The mechanism of this generator is based on the rebounding effect-induced hot carrier transport under the ultrahigh built-in field in the interface, which breaks the symmetry of the drift current and diffusion current in the heterojunction. Furthermore, the output voltage can be delicately designed and enhanced with the interface energy level engineering of inserting various dielectric layers. And hot electrons will still transfer across the interface through the tunneling and hopping effect, under the severely bounding-back and acceleration effect of the large interface electric field. The constant voltage output before and after insertion of a graphene layer at the interface indicates the validity of our unique physical mechanism based on semiconductor physics. Finally, a dynamic black phosphorus/AlN/Si generator with an enhanced voltage output as high as 6.1 V, a current density as high as 124.0 A/m^2^, a power density as high as 201.0 W/m^2^, and an energy-conversion efficiency of 31.4% has been achieved and used to directly and synchronously light up a blue-light LED under continuous mechanical movement excitation. This direct-current generator has the potential to convert ubiquitous mechanical energy into electric energy and is a promising candidate for novel portable and miniaturized power sources in the in situ energy acquisition field.

## 4. Materials and Methods

### 4.1. Device Fabrication

First, a single-side polished Si wafer was dipped into 10 wt% HF for 10 min to remove the native oxide layer on the surface and washed with deionized water. Then, a Ti/Au (10/100 nm) electrode was fabricated with magnetron sputtering on the unpolished side of the Si wafer. The AlN layer was fabricated on the Si substrate using a physical vapor deposition method. The HfO_2_ or Al_2_O_3_ layer was fabricated on the Si substrate using the atomic layer deposition method. A graphene layer was grown on a copper foil using a chemical vapor deposition technique, which could be transferred to the Si substrate using a wet transfer method. The black phosphorus flake was multilayer and stripped by a micromechanical force, and a Ti/Au (10/100 nm) electrode was fabricated with magnetron sputtering on the edge of the black phosphorus flake. The black phosphorus flake was pressed closely onto the Si substrate by external pressure, ensuring that a solid electrical contact between the black phosphorus and Si substrate could be achieved.

### 4.2. Physical Characterization Methods

Microscopic images and the contact area of the generator were obtained via ZEISS optical microscopy. The current voltage (*I*-*V*) curve of the heterojunction was measured with a Keithley 2400 system. The real-time voltage and current output were recorded by a Keithley 2010 or 2600 system, which was controlled by a LabVIEW-based data acquisition system with a sampling rate of 25 s^−1^. The force was measured with a high-precision pressure meter. We designed a customized system to control the force and speed. For the linear mode, the two different types of semiconductors were separately fixed on the desktop and pressure meter. The pressure meter was fixed on a sliding rail and could move horizontally under the control of a microcontroller unit (MCU). There was a driving motor on the sliding rail, which was used to drive the pressure meter. Thus, the semiconductor on the pressure meter could move horizontally and maintain the same moving speed under the control of the MCU. The pressure and speed can be accurately controlled with a fluctuation of 5%. For the circularly rotating working mode, we fixed the driving motor at the edge of the pressure meter, and a mechanical arm was added. The pressure and speed varies in a wider range as the inherent mechanical error such as the flatness of the mechanical arm and frame.

## Supplementary Materials

Supplementary Materials Materials and methods: calculations of Fermi level and energy-conversion efficiency. Figure S1: the lattice of monolayer and multilayer black phosphorus. Figure S2: pictures of experimental system for controlling the applied force and speed of a dynamic heterojunction generator. Figure S3: the schematic diagram of a dynamic black phosphorus/Si heterojunction generator. Figure S4: the temperatures of a 4-inch Si wafer both at the front and back sides before and after sliding. Figure S5: the current output of the dynamic black phosphorus/Si junction generator under dark and light environments. Figure S6: the electrical characteristic analysis of the black phosphorus/AlN/Si heterojunction. Figure S7: pictures taken from a video to show the blue LED powered by our dynamic heterojunction generator.
