# Supplementary material for: Tunable Dynamic Black Phosphorus/Insulator/Si Heterojunction Direct-Current Generator Based on the Hot Electron Transport
Source: Research (Wash D C). 2019 Nov 15;2019:5832382. doi: 10.34133/2019/5832382 (PMC6946282; doi:10.34133/2019/5832382)
Supplement: Supplementary Materials — Materials and methods: calculations of Fermi level and energy-conversion efficiency. Figure S1: the lattice of monolayer and multilayer black phosphorus. Figure S2: pictures of experimental system for controlling the applied force and speed of a dynamic heterojunction generator. Figure S3: the schematic diagram of a dynamic black phosphorus/Si heterojunction generator. Figure S4: the temperatures of a 4-inch Si wafer both at the front and back sides before and after sliding. Figure S5: the current output of the dynamic black phosphorus/Si junction generator under dark and light environments. Figure S6: the electrical characteristic analysis of the black phosphorus/AlN/Si heterojunction. Figure S7: pictures taken from a video to show the blue LED powered by our dynamic heterojunction generator. [file 5832382.f1.docx]

**Supplementary Materials**

**Tunable Dynamic Black Phosphorus/Insulator/Si Heterojunction Direct-Current Generator Based on the Hot Electrons Transport**

*Yanghua Lu^1^, Sirui Feng^1^, Runjiang Shen^1^, Yujun Xu^1^, Zhenzhen Hao^1^, Yanfei Yan^1^, Haonan Zheng^1^, Xutao Yu^1^, Qiuyue Gao^1^, Panpan Zhang^1^ and Shisheng Lin^1,2,*^*

^1^College of microelectronics, College of Information Science and Electronic Engineering, Zhejiang University, Hangzhou, 310027, P. R. China

^2^State Key Laboratory of Modern Optical Instrumentation, Zhejiang University, Hangzhou, 310027, P. R. China

^*^Correspondence: [shishenglin@zju.edu.cn](mailto:shishenglin@zju.edu.cn).

**Supplementary Text:**

**The Fermi level of Si substrate**

The Fermi level of the semiconductor can be calculated by the formula below as below:

$E_{F-N}\approx E_{i}+k_{B}T\ln\frac{n-p}{n_{i}}$ (1)

$E_{F-P}\approx E_{i}-k_{B}T\ln\frac{p-n}{n_{i}}$ (2)

where E_i_ is the middle value of the band gap, k_B_ is the Boltzmann constant, T is the temperature, n is the electron concentration and p is the hole concentration. The n_i_ is the intrinsic carrier concentration of the semiconductor. E_F-N_ and E_F-P_ are the Fermi level of the N-type and P-type semiconductors we used. The hole concentration and intrinsic carrier concentration of the P-type Si substrate used here is 4.34×10^18^ cm^-3^ and 1.5×10^10^ cm^-3^, respectively. The conduction and valence band of P-type Si locate 4.05 eV and 5.17eV below the vacuum energy level, respectively. Therefore, the Fermi level of the P-type Si is calculated as 5.12 eV based on equation (2).

**The energy-conversion efficiency of the dynamic black phosphorus/AlN/Si heterojunction generator**

The working circuit is consisted of a leakage current (I_D_), a series resistance (R_s_), a recombination generated parallel resistance (R_p_) and a load resistance (R_L_). An increasing voltage and decreasing current density can be measured with the increase of load resistance. The power conversion efficiency of the dynamic heterojunction generator can be expressed as below:

$PCE=\frac{P_{max}}{P_{in}}=\frac{V_{oc}\times J_{sc}\times FF}{P_{in}}=\frac{V_{max}\times J_{max}}{P_{in}}=\frac{V_{max}\times J_{max}}{F\times v}$ (3)

$FF=\frac{V_{max}\times J_{max}}{V_{oc}\times J_{sc}}$ (4)

where V_oc_ and J_sc_ are the open-circuit voltage and short-circuit current density of the generator. And V_max_ and J_max_ are the working voltage and current density of the generator under the maximum power output. FF is the ideal factor, F is the [force](file:///C:\Users\Administrator\AppData\Local\youdao\dict\Application\7.5.2.0\resultui\dict\%3fkeyword=force)[of](file:///C:\Users\Administrator\AppData\Local\youdao\dict\Application\7.5.2.0\resultui\dict\%3fkeyword=of)[friction](file:///C:\Users\Administrator\AppData\Local\youdao\dict\Application\7.5.2.0\resultui\dict\%3fkeyword=friction) in the interface, and v is the relative moving speed. V_max_ and J_max_ are calculated with the average working voltage and current density of the direct-current generator in 5.0s. For the dynamic black phosphorus/AlN/Si heterojunction generator in the manuscript, V_oc_ and J_sc_ are as high as 6.1 V and 124.0 A/m^2^. Accordingly, the power density is changed with the electrical load R. Specifically, the peak power output (P_max_) of 201.0 W/m^2^ can be found around R≈450 kΩ, of which the R value is close to the internal resistance (R_s_+R_p_) of the power generation unit. The F between the semiconductors is 0.2 N when the contact area is as large as 0.25 cm^2^. The moving speed is 8.0 cm/s here, so the energy-conversion efficiency of the dynamic black phosphorus/AlN/Si heterojunction generator can be calculated to be about 31.41%.

**Supplementary Figure:**


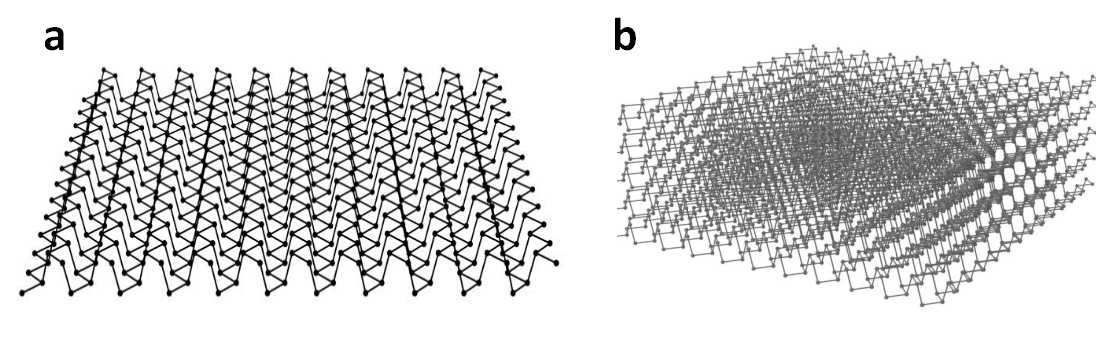


**Figure S1:** The lattice of monolayer and multilayer black phosphorus.


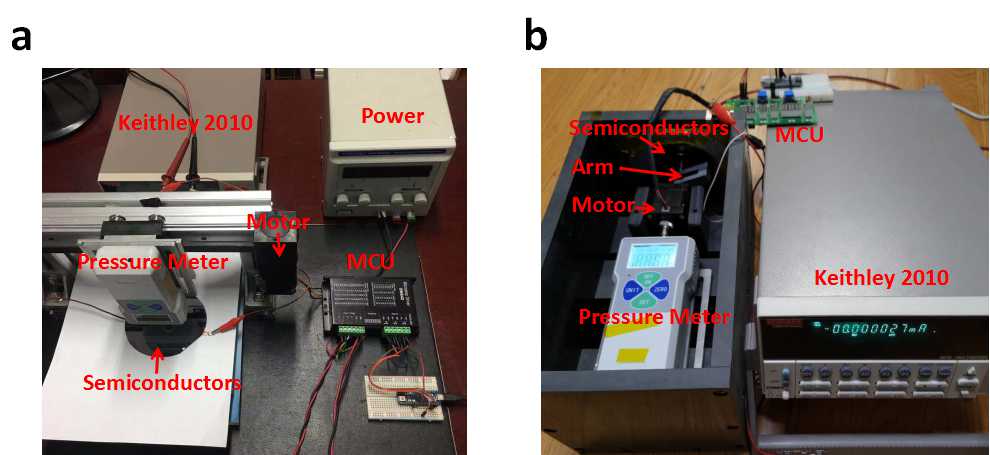


**Figure S2:** Experimental designed system for controlling the applied force and speed of dynamic heterojunction generator.


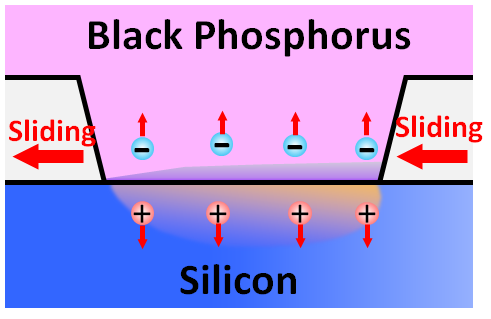


**Figure S3:** The schematic diagram of dynamic black phosphorus/Si heterojunction generator.

**
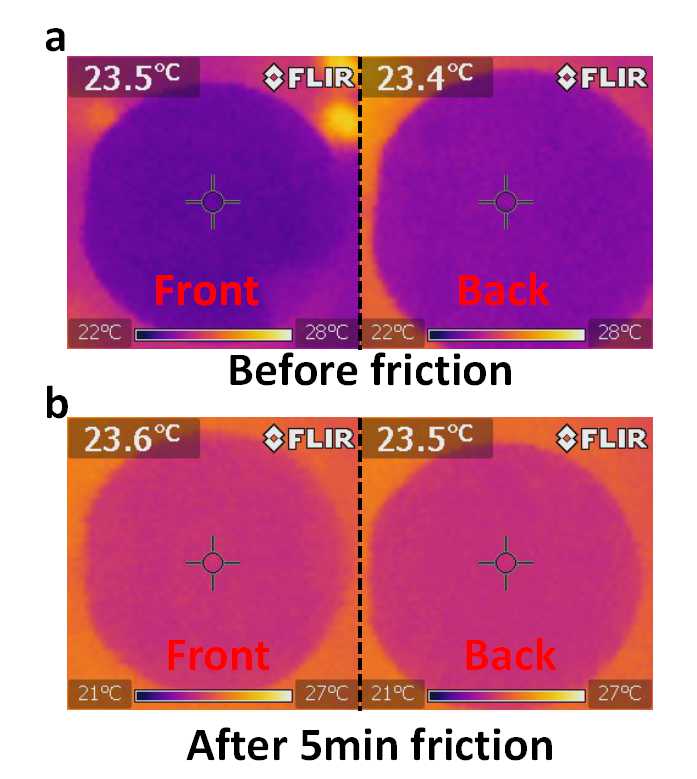
**

**Figure S4:** The temperatures of 4-inch Si wafer both the front and back side before and after sliding.


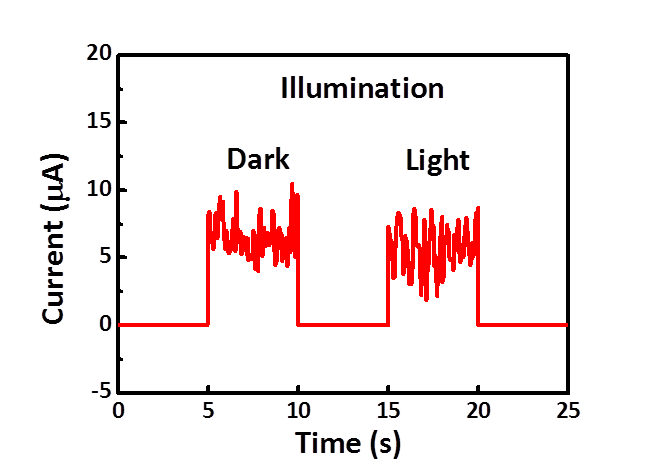


**Figure S5:** The current output of the dynamic black phosphorus/Si junction generator under dark and light environment.


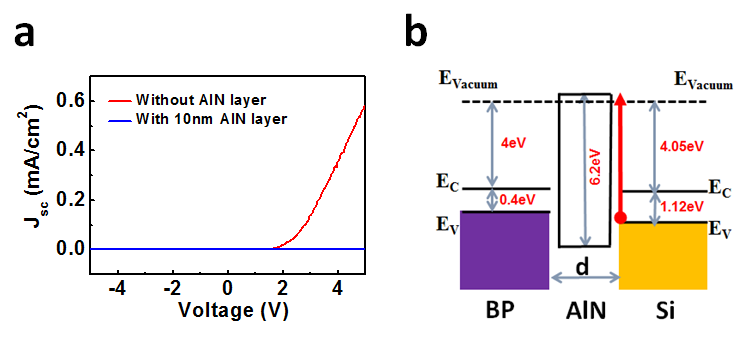


**Figure S6:** The electrical characteristic analysis of the black phosphorus/AlN/Si heterojunction. (a) Current density-voltage curves of black phosphorus/Si heterojunction with and without 10 nm AlN layer. (b) The one-dimensional band diagram of black phosphorus/AlN/Si heterojunction.

*
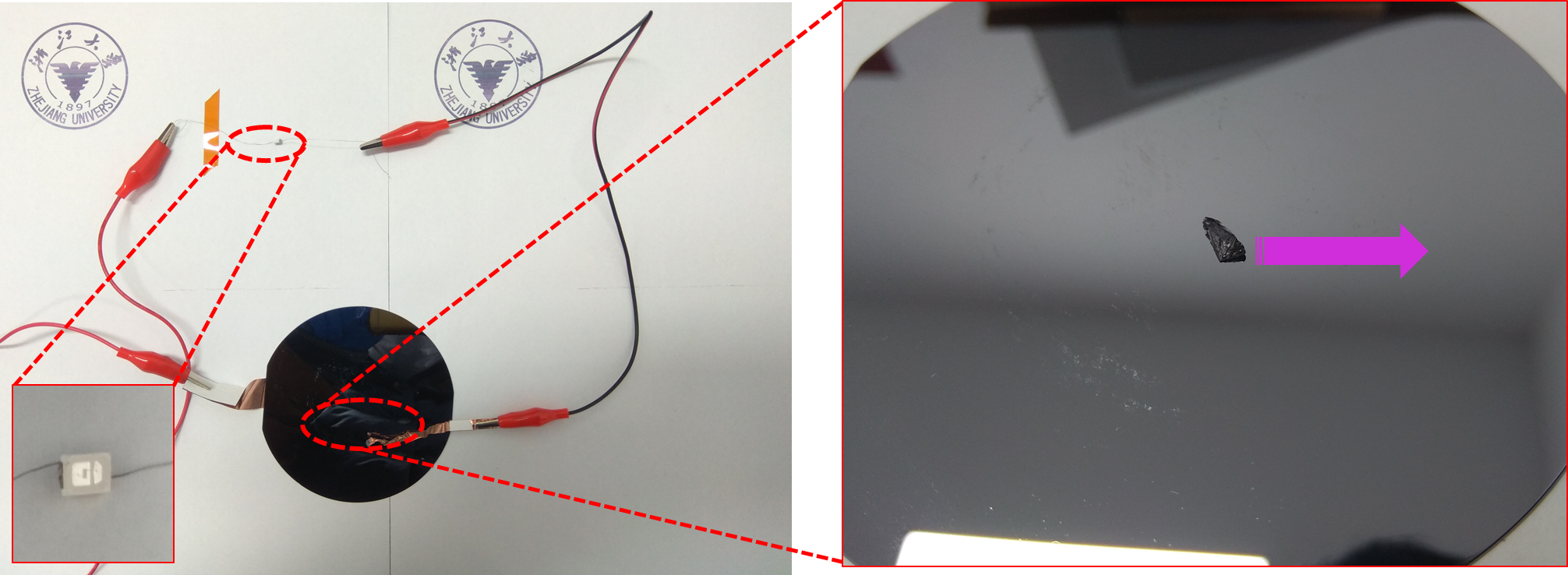
*

**Figure S7:** Pictures taken from video to show the blue LED powered by our dynamic heterojunction generator.
